# Inhibition among olfactory receptor neurons

**DOI:** 10.3389/fnhum.2013.00690

**Published:** 2013-10-23

**Authors:** Wynand Van der Goes van Naters

**Affiliations:** School of Biosciences, Cardiff UniversityCardiff, UK

**Keywords:** olfaction, inhibition, ephaptic, sensillum, sensory neurons, *Drosophila*

## Abstract

Often assumed to be epiphenomena of a cell’s activity, extracellular currents and resulting potential changes are increasingly recognized to influence the function of other cells in the vicinity. Experimental evidence shows that even small electric fields can modulate spike timing in neurons. Moreover, when neurons are brought close together experimentally or in pathological conditions, activity in one neuron can excite its neighbors. Inhibitory ephaptic mechanisms, however, may depend on more specialized coupling among cells. Recent studies in the *Drosophila* olfactory system have shown that excitation of a sensory neuron can inhibit its neighbor, and it was speculated that this interaction was ephaptic. Here we give an overview of ephaptic interactions that effect changes in spike timing, excitation or inhibition in diverse systems with potential relevance to human neuroscience. We examine the mechanism of the inhibitory interaction in the *Drosophila* system and that of the well-studied ephaptic inhibition of the Mauthner cell in more detail. We note that both current towards and current away from the local extracellular environment of a neuron can inhibit it, but the mechanism depends on the specific architecture of each system.

While chemical and electrical synapses are anatomically identifiable specialized contacts that mediate interactions among cells, electric fields and currents produced by cells in their local environment may also influence the activity of their neighbors (Jefferys, 1995; Anastassiou et al., 2011). The extent to which these ephaptic interactions contribute to information processing in the human nervous system is not yet clear. Our understanding of ephaptic mechanisms has been especially informed by investigating the communication among cells in several very tractable systems, including the Mauthner cells in fish (Furukawa and Furshpan, 1963; reviewed in Zottoli and Faber, 2000; Korn and Faber, 2005; Weiss and Faber, 2010). Recently, Su et al. (2012) showed that inhibitory interactions among grouped olfactory sensory neurons in the fruit fly *Drosophila melanogaster* modulate their responses to odors, suggesting that processing of olfactory information may already begin among neurons within the sensory organs. Blocking synaptic transmission by several experimental means did not remove the inhibition, which together with other considerations led the authors to hypothesize that an ephaptic mechanism underlies the interaction. Here we present a brief overview of ephaptic interactions in diverse systems and then focus on inhibitory ephaptic mechanisms in the Mauthner cell system and in the *Drosophila* olfactory system.

The transmembrane potential (*V*_m_) of a cell is the difference between the electrical potential inside the cell (*V*_i_) and the potential externally (*V*_e_), which are both measured against the same (distant) reference point, such that *V*_m_ = *V*_i_−*V*_e_. The resting *V*_m_ in many neurons is approximately −70 mV. While *V*_e_ is often assumed to be constant and assigned a value of 0 V by convention, current flow to or from the local extracellular environment near the cell can change local *V*_e_ and thereby *V*_m_. The direction of electric current is defined arbitrarily as if only positive charges are moving, so in the case of movement of anions the current is opposite in direction to the actual anionic flow. Current flow to the local extracellular environment may hyperpolarize the membrane by making *V*_e_ more positive, while current flow away may reduce the absolute potential difference between *V*_i_ and *V*_e_. Current flow is the result of an electric field, which is defined as the force per unit positive charge acting on a charged particle and is expressed in newtons per coulomb or volts per meter (N/C = V/m). A field can act to drive current in the extracellular space and also across the membrane and intracellularly.

Even very small fields can change the timing of spikes generated by neurons, as was shown in rat cortical pyramidal neurons where changes under 0.2 mV in *V*_e_ induced by an external oscillating field could entrain spikes (Anastassiou et al., 2011). Small electric fields, when concurrent to other suprathreshold input, also had significant effects in rat hippocampal neurons on spike timing and these could be magnified by dynamic network activity (Radman et al., 2007; Reato et al., 2010). It is becoming increasingly clear that, in addition to the firing rate of an individual neuron, its precise spike timing in an ensemble of neurons in a circuit has an important role in relaying information (De Zeeuw et al., 2011). Moreover, the degree of phase-locking of hippocampal neurons to the theta-oscillation was shown to be predictive for the strength of human memory formation (Rutishauser et al., 2010), indicating a role for spike timing in information storage. The ability of electric fields to affect spike timing suggests ephaptic interactions may indeed contribute to information processing in the brain in combination with other intercellular mechanisms. Ephaptic effects on spike timing may also play a role in the pathological transition from synchronous discharge into epileptic seizure in several brain areas (Jefferys, 1995; Vigmond et al., 1997; McCormick and Contreras, 2001).

In addition to modulating the timing of spikes, stronger fields can bring neurons to threshold, especially under experimental or pathological conditions. Indeed, early studies showed that action potentials could be transmitted between two experimentally apposed squid giant axon fibers (Arvanitaki, 1942; Ramón, and Moore, 1978) and it was Arvanitaki (1942) who first called the zone of contact or close vicinity, where the cells can influence each other’s activity, an ephapse. Clinically, the sudden sharp pains experienced by patients with trigeminal neuralgia may in part be caused by ephaptic neurotransmission between apposed denuded axonal membranes of nociceptors near the trigeminal ganglion (Oaklander, 2008). Similarly, the involuntary contractions of facial muscles in hemi-facial spasm have been attributed to ephapsis among fibers in the facial nerve when it is chronically but often subclinically injured by pressure of a blood vessel, although mechanisms such as ectopic generation of muscle discharges may also contribute (Valls-Solé, 2013). Computational approaches suggest that close apposition, as occurs among the tightly packed fascicles of unmyelinated axons of olfactory receptor neurons, also allows an action potential in one fiber to elicit action potentials in its neighbors under normal physiological conditions through ephaptic coupling (Bokil et al., 2001), so even in the absence of gap junctions between them (Blinder et al., 2003).

In contrast to excitatory coupling, ephaptic inhibition among cells may require specialized anatomical, molecular and electrical features. In the retina, for example, connexin hemichannels may be involved in an ephaptic negative feedback mechanism whereby voltage changes in horizontal cells result in sign-inverted changes in the cone cells (Klaassen et al., 2012). In the cerebellum Purkinje cells receive inhibitory input from basket cells whose axons establish chemical synapses on the Purkinje soma but also ramify around the axon initial segment of Purkinje cells forming a highly organized structure, the pinceau. Electrophysiological investigation showed that a field effect inhibition is exerted on the axon initial segment (Korn and Axelrad, 1980). Indeed there are few direct chemical synapses from basket cell axons on the Purkinje cells within the pinceau (see e.g., Somogyi and Hámori, 1976), and several GABAergic signaling components show only low expression in it (Iwakura et al., 2012). The pinceau may therefore mediate ephaptic modulation by basket cells of Purkinje activity in a similar way as the well-studied axon cap that holds the terminals of interneurons around the initial segment of the Mauthner cell.

The Mauthner (or “M−”) cells are a pair of neurons in the hindbrain of teleosts and amphibians that integrate auditory, visual and tactile sensory inputs to generate an escape reflex of the animal, the C-start (Zottoli and Faber, 2000; Korn and Faber, 2005). The descending axon of a Mauthner cell crosses over to the contralateral side. Sensory inputs collected on two main dendritic branches bring the Mauthner cell to threshold and an action potential, initiated at or near the axon hillock on a proximal unmyelinated axonal segment, propagates along the axon to contract the musculature on the contralateral side of the body (Figure 1A). Fast inhibitory feedback and feed forward through interneurons limits activation of the Mauthner cell to a single spike and stops simultaneous excitation of the other Mauthner cell, which together results in the characteristic C-shaped whipping movement of the body that displaces the animal away from the triggering stimuli. The fast inhibition arrives at the same time as the Mauthner cell receives excitatory input, suggesting that the inhibition contributes to setting the threshold of the startle response (Weiss et al., 2008). An ephaptic mechanism underlies the fast inhibition (Furukawa and Furshpan, 1963). The axons of specific interneurons terminate near the axon hillock of the Mauthner cell in a highly organized structure, the axon cap. The axon terminals are unmyelinated and, on excitation of the interneurons, inward currents at the last node of Ranvier cause current to flow out from the axon terminals into the local environment of the Mauthner cell. High electrical resistance in the axon cap causes the current flow into it to locally increase *V*_e_ around part of the Mauthner cell. While leakage across the Mauthner membrane also causes a slight increase in *V*_i_, simultaneous measurements of *V*_e_ and *V*_i_ have shown that the increase in *V*_e_ is much larger suggesting that the potential increase is indeed due to current from an extrinsic source. In the Mauthner system, a current from the axon terminals of interneurons to the environment around part of the Mauthner cell thereby results in a local extrinsic hyperpolarizing potential (EHP) that inhibits the cell’s activity. Reciprocal ephaptic inhibition also occurs. An action potential in the Mauthner cell leads to a passive hyperpolarizing potential (PHP) in the interneurons innervating the axon cap, which has been used experimentally to identify them, because the action current leaves the Mauthner cell at its soma and dendrites and its extracellular return path brings part of the current to the excitable region of these PHP interneurons (Faber and Korn, 1989); the momentary inhibition produced by the PHP is thought to be essential for subsequently synchronizing these interneurons for feedback inhibition.

**Figure 1:**
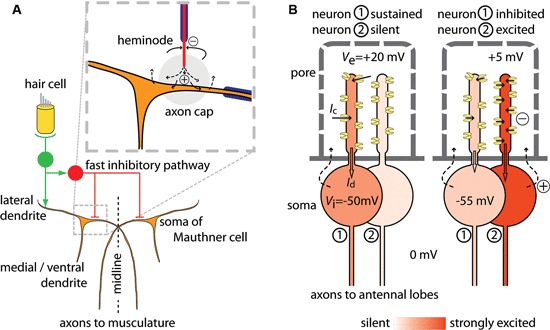
**Schematic of currents that mediate ephaptic inhibition in the Mauthner cell and in *Drosophila* olfactory receptor neurons.** **(A)** A pair of Mauthner cells (in brown) project axons to the contralateral side of the body (redrawn and adapted from Korn and Faber, 2005; Weiss et al., 2008). Only the part of the circuit that mediates fast excitation and fast ephaptic inhibition from hair cell input is shown. Excitation of VIII nerves (in green) by hair cell input excites both the ipsilateral Mauthner cell and inhibitory interneurons (in red) through mixed electrical and chemical synapses. The fast inhibition by the interneurons acts on both Mauthner cells. Axons of these neurons terminate in the axon cap, a structure of high resistivity (enlarged inset, grey circle). Current influx at the heminode flows out at the unmyelinated axon terminal within the axon cap, thereby increasing the extracellular potential *V*_*e*_ and hyperpolarizing the zone of the Mauthner cell where impulse initiation occurs. **(B)** A *Drosophila* sensillum with two neurons. The sensillum hair has pores through which odor molecules enter. The hair lumen forms a compartment holding the dendrites of the two neurons. At left, neuron 1 shows a sustained response to odor and neuron 2 is silent. An odorant causes channels to open in neuron 1 and each open channel carries a current *I*_*c*_, which sum to the dendritic current *I*_*d*_ (block arrow) that depolarizes the soma region. At right, neuron 1 is inhibited by the transient excitation of neuron 2. Opening of many channels in neuron 2 draws current from the extracellular dendritic space, which reduces *V*_*e*_ also for neuron 1. The per-channel current *I*_*c*_ decreases, so that *I*_*d*_ in neuron 1 also decreases and the soma becomes less depolarized. Illustrative values of potentials are given for *V*_*i*_ and *V*_*e*_. The return source current (dashed arrows) follows a complex path, and also involves auxiliary cells (not shown). For both **(A)** and **(B)**: currents from sources are shown as dashed arrows; currents to sinks are shown as solid arrows; sources and sinks are defined as in Buzsáki et al. (2012).

In *Drosophila*, olfactory receptor neurons in the antennae group into units called sensilla (Shanbhag et al., 1999, 2000). The response to odorants can be excitatory, inhibitory or a sequential combination of excitation and inhibition. Recently it was found that the sustained action potential response of one neuron is inhibited by a transient excitation of a neighbor within the sensillum and, based on experiments which suggested the interaction was non-synaptic, an ephaptic mechanism was proposed (Su et al., 2012). The neurons in a sensillum have different odor specificities (De Bruyne et al., 2001). Neurons express one, or sometimes more than one (Goldman et al., 2005), odor receptor. Each receptor confers specificity to a narrow or a broader range of different food- and other environmental odors (Dobritsa et al., 2003; Hallem and Carlson, 2006; Benton et al., 2009; Ai et al., 2010; Stensmyr et al., 2012) or to fly odors that may act as pheromones (Van der Goes van Naters and Carlson, 2007). Sensilla are externally visible as hairs or pegs with many pores (Figure 1B) through which the odor molecules from the air can enter. The hair lumen forms a compartment that holds the dendrites of the olfactory receptor neurons. As is the case in other cells that are embedded in epithelia, the neurons contact a different milieu apically than basally. The dendritic processes of the olfactory neurons sharing a sensillum are bathed together in lymph that is high in [K^+^] and low in [Na^+^] (in moth sensilla: Kaissling and Thorson, 1980), reminiscent of the apical environment of hair cells in the inner ear, while their somata are surrounded by fluid resembling other extracellular fluids that are low in [K^+^] and high in [Na^+^]. The external potential *V*_e_ around the dendrites is approximately 30–35 mV higher than the *V*_e_ of the soma. This external potential around the dendrites decreases during an odor stimulus, because channel opening allows current to flow from the lymph compartment into the dendrites. The current from the lymph into the dendrite is then conducted proximally and depolarizes the soma’s membrane to generate action potentials. In this model, the rate at which the neuron generates action potentials is determined by the size of the current coming from the dendritic compartment into the soma region. The size of the inward dendritic current depends on the number of open channels, which is determined by the odor stimulus, and the amount of current per channel, which is affected by *V*_e_. A transient decrease in *V*_e_ caused by an odor stimulus for a neighboring neuron will reduce the current through each open channel and also the dendritic current into the soma region. This decrease in the depolarizing current will then slow the generation of action potentials by the neuron. A straightforward test for this mechanism could be achieved by connecting two sensilla with a wire. In this configuration we would predict that the sustained response of a neuron in one sensillum could be inhibited by excitation of a neuron in the connected sensillum. Inhibition in this configuration would show that the interaction can be explained by current flow through the extracellular space.

In comparing one aspect of the Mauthner cell system and the *Drosophila* sensillum, we note that ephaptic inhibition of a neuron can occur both by current flow to and by current flow from its local environment. In the Mauthner cell system, current towards the axon hillock increases local *V*_e_ and thereby hyperpolarizes the excitable part of the membrane. In the *Drosophila* sensillum, by contrast, inhibition is caused by current flow out from the dendritic environment of a neuron showing a sustained response. While current flow from the local environment causes a decrease in *V*_e_ and thereby a depolarization of the dendritic membrane, this local change in membrane potential does not affect the neuron’s firing rate directly because the dendrite is presumably non-excitable. Rather, the drop in *V*_e_ causes a decrease in the per-channel current and thereby reduces the dendritic current into the soma region. This results in an inhibition of the neuron’s action potential activity. The time course of inhibition appears to mirror the kinetics of excitation of its neighbor (data in Su et al., 2012). In both systems, the ephaptic interaction is dependent on compartmentalization of the extracellular space so that local current can significantly change *V*_e_.

The function of the inhibition of a sustained response of one neuron in a *Drosophila* sensillum by transient activation of another neuron may be to increase salience of the odor transient (Su et al., 2012). Compartmentalization of groups of sensory cells, as occurs in the *Drosophila* olfactory sensillum, is found in a number of other systems including the mammalian taste bud. While the focus of much research has been on stimulus-response characteristics of individual sensory cells, it is not yet understood how the cells in a group modulate each other’s output. The inhibitory interaction found in *Drosophila* suggests initial processing of information starts at the periphery, and that ephaptic interactions may play an important role.

It is becoming increasingly clear that ephaptic interactions make an essential contribution to information processing in the nervous system. The rapid kinetics by which cell function can be modulated through electric fields suggest ephaptic interactions may act especially in a short time domain to complement slower intercellular communication through chemical synapses. Elucidating the mechanisms that operate in small circuits, such as the Mauthner cell system and the *Drosophila* olfactory sensillum, will be essential to understand processing in more complex networks.

## Conflict of interest statement

The authors declare that the research was conducted in the absence of any commercial or financial relationships that could be construed as a potential conflict of interest.
